# Supplementary material for: Phenotypic and Genotypic Analysis of Antimicrobial Resistance in Escherichia coli Recovered from Feedlot Beef Cattle in Australia
Source: Animals (Basel). 2022 Aug 31;12(17):2256. doi: 10.3390/ani12172256 (PMC9454709; doi:10.3390/ani12172256)
Supplement: Supplementary file 1 [file animals-12-02256-s001.zip › animals-1880130-supplementary.pdf]

Table S1. The antimicrobial resistance pattern of *E. coli* isolated from antimicrobial treated and non-treated cattle at feedlot exit

	Total no. of isolates (%)		Resistance pattern (no. of isolates)	
**Number of antimicrobial classes**	**Non-treated (122)**	**Treated (13)**	**Non-treated**	**Treated**
All sensitive	93 (76.23)	10 (76.9)	93	10
1	15 (12.29)	2 (15.4)	TET (12)	TET (1)(***)
			FIS (1)	AMP-AUG2-AXO-FOX-XNL (1)[a](**)
			AMP (1)	
			AUG2-FOX (1)	
2	4 (3.28)		AMP-TET (3)	
			FIS-TET (1)	
3	7 (5.74)		FIS-STR-TET (3)	
			AMP-STR-TET(3)	
			AMP-AXO-AZI-TET-XNL (1)[a]	
5	3 (2.46)	1 (7.7)	AMP-AXO-AZI-FIS-STR-TET-XNL (2)[a]	AMP-AXO-FIS-STR-SXT-TET-XNL (1)[a](*)
			AMP-AXO-CHL-FIS-STR-SXT-TET-XNL (1)[a]	
Resistance (%)	29 (23.8)	3 (23.1)		
P-value	0.955			

[a]ESBL producing *E.coli*; *Draxxin Tulathromycin); **Excede (Ceftiofur); ***Bivatop (Oxytetracycline); **AUG** (Amoxicillin/clavulanic acid), **AMP** (Ampicillin), **FOX** (Cefoxitin), **XNL** (Ceftiofur), **AXO** (Ceftriaxone), **CHL** (Chloramphenicol), **STR** (Streptomycin), **FIS** (Sulfisoxazole), **TET** (Tetracycline) and **SXT** (Trimethoprim/sulfamethoxazole)

Table S2. Agreement between antimicrobial resistance phenotypes and resistance gene detection among 37 isolates submitted for WGS

**Isolates**	**Phenotypic AMR pattern**	**Genotypic AMR pattern**
N001	Susceptible	-
N002	Susceptible	-
N308	Susceptible	-
N309	Susceptible	-

N338	FIS	*sul2*
N344	AMP	*bla*$_{TEM-1B}$
N330	AMP-AUG-AXO-FOX-XNL	*bla*$_{CMY-2}$
N305	TET	*tet(B)*
N314	TET	*tet(B)*
N316	TET	*tet(B)*
N348	TET	*tet(A)*
N351	TET	*tet(B)*
N352	TET	*tet(B)*
N355	TET	*tet(A)*
N396	TET	-
N401	TET	*tet(B)*
N413	TET	*tet(B)*
N415	TET	*tet(A)*
N435	TET	*tet(B)*
N442	TET	*tet(A)*
N321	AMP-TET	*bla*$_{TEM-1C}$, *tet(A)*
N358	AMP-TET	*bla*$_{TEM-1C}$, *tet(A)*
N381	AMP-TET	*aph(3'')-Ib*, *aph(6)-Id*, *bla*$_{TEM-1B}$, *tet(B)*
N385	FIS-TET	*sul2*, *tet(B)*
N112	AMP-AUG-AXO-FOX-TET-XNL	*bla*$_{CMY-2}$, *bla*$_{TEM-1B}$, *dfrA5*, *tet(A)*
N324	AMP-STR-TET	*aph(3'')-Ib*, *aph(6)-Id*, *bla*$_{TEM-1B}$, *tet(B)*
N333	AMP-STR-TET	*aph(3'')-Ib*, *aph(6)-Id*, *bla*$_{TEM-1B}$, *tet(B)*
N341	AMP-STR-TET	*aph(3'')-Ib*, *aph(6)-Id*, *bla*$_{TEM-1B}$, *tet(B)*
N054	AMP-STR-SXT	*aph(3')-Ia*, *aph(3'')-Ib*, *aph(6)-Id*, *bla*$_{TEM-1B}$, *dfrA5*, *sul2*
N369	FIS-STR-TET	*aph(3'')-Ib*, *aph(6)-Id*, *sul2*, *tet(B)*
N373	FIS-STR-TET	*aph(3'')-Ib*, *aph(6)-Id*, *sul2*, *tet(B)*
N443	FIS-STR-TET	*aph(3'')-Ib*, *aph(6)-Id*, *sul2*, *tet(B)*
N325	AMP-AXO-AZI-TET-XNL	*bla*$_{CTX-M-15}$, *mph(A)*, *mph(E)*, *msr(E)*, *tet(A)*
N423	AMP-AXO-FIS-STR-SXT-TET-XNL	*aph(3'')-Ib*, *aph(6)-Id*, *bla*$_{CTX-M-15}$, *bla*$_{TEM-1B}$, *dfrA14*, *sul2*, *tet(A)*
N307	AMP-AXO-AZI-FIS-STR-TET-XNL	*aph(3'')-Ib*, *aph(6)-Id*, *bla*$_{CTX-M-27}$, *mph(A)*, *sul2*, *tet(A)*
N347	AMP-AXO-AZI-FIS-STR-TET-XNL	*aph(3'')-Ib*, *aph(6)-Id*, *bla*$_{CTX-M-27}$, *mph(A)*, *sul2*, *tet(A)*

N354	AMP-AXO-CHL-FIS-STR-TET-XNL	*aac(3)-IV,ant(3′′)-Ia,aph(3′′)-Ib, aph(4)-Ia, aph(6)-Id, bla*$_{CTX-M-15}$, *bla*$_{TEM-1B}$, *cmlA1, dfrA12, qnrS1, sul1, sul3, tet(A)*

Table S3. Correlation analysis of phenotypic and genotypic AMR in *E.coli* isolated from feedlot and abattoir

Variables	AMP_P	AUG_P	AXO_P	AZI_P	CHL_P	FIS_P	FOX_P	STR_P	SXT_P	TET_P	XNL_P	AMP_G	AUG_G	AXO_G	AZI_G	CHL_G	FIS_G	FOX_G	STR_G	SXT_G	TET_G	XNL_G
AMP_P	1.000																					
AUG_P	0.289	1.000																				
	(0.082)																					
AXO_P	0.585*	0.495*	1.000																			
	(0.000)	(0.002)																				
AZI_P	0.360*	-0.071	0.615*	1.000																		
	(0.029)	(0.676)	(0.000)																			
CHL_P	0.202	-0.040	0.345*	-0.050	1.000																	
	(0.231)	(0.815)	(0.036)	(0.771)																		
FIS_P	0.045	-0.136	0.369*	0.293	0.294	1.000																
	(0.791)	(0.424)	(0.024)	(0.078)	(0.077)																	
FOX_P	0.289	1.000*	0.495*	-0.071	-0.040	-0.136	1.000															
	(0.082)	(0.000)	(0.002)	(0.676)	(0.815)	(0.424)																

STR_P	0.426*	-0.155	0.290	0.240	0.256	0.596*	-0.155	1.000						
	(0.008)	(0.358)	(0.082)	(0.152)	(0.126)	(0.000)	(0.358)							
SXT_P	0.360*	-0.071	0.362*	-0.088	0.561*	0.293	-0.071	0.457*	1.000					
	(0.029)	(0.676)	(0.028)	(0.604)	(0.000)	(0.078)	(0.676)	(0.004)						
TET_P	0.033	-0.165	0.086	0.156	0.088	0.145	-0.165	0.198	-0.085	1.000				
	(0.848)	(0.330)	(0.612)	(0.356)	(0.606)	(0.393)	(0.330)	(0.240)	(0.619)					
XNL_P	0.585*	0.495*	1.000*	0.615*	0.345*	0.369*	0.495*	0.290	0.362*	0.086	1.000			
	(0.000)	(0.002)	(0.000)	(0.000)	(0.036)	(0.024)	(0.002)	(0.082)	(0.028)	(0.612)				
AMP_G	1.000*	0.289	0.585*	0.360*	0.202	0.045	0.289	0.426*	0.360*	0.033	0.585*	1.000		
	(0.000)	(0.082)	(0.000)	(0.029)	(0.231)	(0.791)	(0.082)	(0.008)	(0.029)	(0.848)	(0.000)			
AUG_G	0.289	1.000*	0.495*	-0.071	-0.040	-0.136	1.000*	-0.155	-0.071	-0.165	0.495*	0.289	1.000	
	(0.082)	(0.000)	(0.002)	(0.676)	(0.815)	(0.424)	(0.000)	(0.358)	(0.676)	(0.330)	(0.002)	(0.082)		
AXO_G	0.585*	0.495*	1.000*	0.615*	0.345*	0.369*	0.495*	0.290	0.362*	0.086	1.000*	0.585*	0.495*	1.000
	(0.000)	(0.002)	(0.000)	(0.000)	(0.036)	(0.024)	(0.002)	(0.082)	(0.028)	(0.612)	(0.000)	(0.000)	(0.002)	

AZI_G	0.360*	-0.071	0.615*	1.000*	-0.050	0.293	-0.071	0.240	-0.088	0.156	0.615*	0.360*	-0.071	0.615*	1.000						
	(0.029)	(0.676)	(0.000)	(0.000)	(0.771)	(0.078)	(0.676)	(0.152)	(0.604)	(0.356)	(0.000)	(0.029)	(0.676)	(0.000)							
CHL_G	0.202	-0.040	0.345*	-0.050	1.000*	0.294	-0.040	0.256	0.561*	0.088	0.345*	0.202	-0.040	0.345*	-0.050	1.000					
	(0.231)	(0.815)	(0.036)	(0.771)	(0.000)	(0.077)	(0.815)	(0.126)	(0.000)	(0.606)	(0.036)	(0.231)	(0.815)	(0.036)	(0.771)						
FIS_G	0.117	-0.145	0.328*	0.265	0.274	0.932*	-0.145	0.669*	0.488*	0.024	0.328*	0.117	-0.145	0.328*	0.265	0.274	1.000				
	(0.489)	(0.390)	(0.048)	(0.113)	(0.101)	(0.000)	(0.390)	(0.000)	(0.002)	(0.888)	(0.048)	(0.489)	(0.390)	(0.048)	(0.113)	(0.101)					
FOX_G	0.289	1.000*	0.495*	-0.071	-0.040	-0.136	1.000*	-0.155	-0.071	-0.165	0.495*	0.289	1.000*	0.495*	-0.071	-0.040	-0.145	1.000			
	(0.082)	(0.000)	(0.002)	(0.676)	(0.815)	(0.424)	(0.000)	(0.358)	(0.676)	(0.330)	(0.002)	(0.082)	(0.000)	(0.002)	(0.676)	(0.815)	(0.390)				
STR_G	0.486*	-0.166	0.255	0.217	0.241	0.549*	-0.166	0.939*	0.429*	0.224	0.255	0.486*	-0.166	0.255	0.217	0.241	0.618*	-0.166	1.000		
	(0.002)	(0.327)	(0.128)	(0.197)	(0.152)	(0.000)	(0.327)	(0.000)	(0.008)	(0.183)	(0.128)	(0.002)	(0.327)	(0.128)	(0.197)	(0.152)	(0.000)	(0.327)			
SXT_G	0.422*	0.302	0.499*	-0.103	0.479*	0.208	0.302	0.345*	0.853*	-0.029	0.499*	0.422*	0.302	0.499*	-0.103	0.479*	0.376*	0.302	0.317	1.000	
	(0.009)	(0.070)	(0.002)	(0.542)	(0.003)	(0.216)	(0.070)	(0.037)	(0.000)	(0.867)	(0.002)	(0.009)	(0.070)	(0.002)	(0.542)	(0.003)	(0.022)	(0.070)	(0.056)		
TET_G	0.083	-0.143	0.113	0.168	0.094	0.175	-0.143	0.231	-0.062	0.926*	0.113	0.083	-0.143	0.113	0.168	0.094	0.061	-0.143	0.258	-0.005	1.000

	(0.624)	(0.398)	(0.505)	(0.319)	(0.578)	(0.301)	(0.398)	(0.169)	(0.714)	(0.000)	(0.505)	(0.624)	(0.398)	(0.505)	(0.319)	(0.578)	(0.718)	(0.398)	(0.123)	(0.974)		
XNL_G	0.585*	0.495*	1.000*	0.615*	0.345*	0.369*	0.495*	0.290	0.362*	0.086	1.000*	0.585*	0.495*	1.000*	0.615*	0.345*	0.328*	0.495*	0.255	0.499*	0.113	1.000
	(0.000)	(0.002)	(0.000)	(0.000)	(0.036)	(0.024)	(0.002)	(0.082)	(0.028)	(0.612)	(0.000)	(0.000)	(0.002)	(0.000)	(0.000)	(0.036)	(0.048)	(0.002)	(0.128)	(0.002)	(0.505)	

* *p<0.05*
